# Did Economic Crisis Affect Mortality Due to Infectious Diseases? Trends of Infectious Diseases Mortality in Greece Before and After Economic Crisis

**DOI:** 10.7759/cureus.13621

**Published:** 2021-02-28

**Authors:** Christos Zilidis, Dimitrios Papagiannis, Zacharoula Kyriakopoulou

**Affiliations:** 1 Epidemiology and Social Medicine, University of Thessaly, Larissa, GRC; 2 Nursing, University of Thessaly, Larissa, GRC; 3 Microbiology, University of Thessaly, Larissa, GRC

**Keywords:** infectious diseases, mortality, economic crisis, austerity, social determinants, greece

## Abstract

Introduction

Economic crisis and the restrictive policies applied in Greece and other countries raise questions about whether financial crises may affect the declining trend of infectious diseases. The aim of this study is to explore the impact of the economic crisis on mortality due to infectious diseases in Greece and its possible correlation with socio-economic variables affected by the crisis.

Methods

Data including all deaths due to infectious diseases in Greece during 2001-2016 were analyzed. Annual total and cause-specific standardized death rates (SDR) and age-specific mortality rates were calculated. Cumulative SDRs and standardized rate ratios of the exposed and the non-exposed to austerity periods were computed. The correlation of mortality with Gross Domestic Product (GDP), unemployment, long-term unemployment and hospital expenditure was explored.

Results

During the exposed-to-austerity period, the SDR of infectious diseases recorded a significant increase by 5% (2.4%-7.7%), exhibiting different trends in the various groups of diseases. The cause-specific SDR increased significantly in intestinal infections, viral diseases, pneumonia, and influenza, and declined in tuberculosis and meningitis. Overall mortality was positively correlated with GDP and unemployment, and adversely with hospital expenditure.

Conclusions

The mortality of infectious disease was adversely affected by economic crisis and austerity, but the effects were found disease-dependent, with significant differences between the various groups of infectious disease. Unemployment and hospital expenditure were the main socio-economic determinants of mortality. Causal mechanisms of the impact remain unclear, requiring further research.

## Introduction

During the past decades, mortality due to infectious diseases declined worldwide [1]. However, the economic crisis of 2008 and the subsequent restrictive policies applied in Greece and several European countries raise questions about whether the crisis may affect this declining trend [2]. The new round of global recession following the COVID-19 pandemic brought back in discussion these questions. In many countries, the financial crisis imposed austerity measures affecting public health, health system performance, and pharmaceutical care [3], raising concern about whether these effects may affect the overall control of infectious diseases [4]. In Greece, the health effects of the financial crisis became more visible after 2010 [5], with a rise in mortality of certain conditions, though a downwards trajectory in others [6]. Several studies suggest that economic crises may increase transmission of communicable diseases [7], affect drug treatment and microbial resistance [8], or lead to neglect of vaccination [9], while other studies suggest that crisis may result in a rise in mortality among specific population groups, such as children [9,10], unemployed [7], migrants [11], or intravenous drug users [12]. Focusing on the effects on mortality of the general population, studies from various European countries had divergent findings. Certain studies found that mortality of infectious diseases increased during the crisis [13,14], while others observed a reduction [15] or no effect [16]. Some of these studies explored the association of mortality with the affected socio-economic variables [13,15,16], while others did not [14]. Based on the existing evidence, it remains unclear whether the potential impact can affect mortality rates [7], or it is under the threshold of producing countable effects.

The main characteristics of the financial crisis in Greece are the reduction of almost 25% of GDP in a few years, the vertical increase in unemployment from 8% to about 25%, and the prolongation of the effects over a long period. This means that the effects on health and healthcare are a result of longer exposure to adverse socio-economic effects and austerity policies. In that context, the aim of this study is to explore the trends of mortality due to infectious diseases in Greece before and after the economic crisis onset, to investigate whether austerity measures had an impact on mortality trends, and to explore possible correlations of mortality trends with the main socio-economic variables affected by the financial crisis.

## Materials and methods

Study design

A cross‐sectional design was applied to compare standardized mortality rates of infectious diseases between the exposed and the non-exposed to the economic crisis period in Greece.

Data sources

The study was based on data extracted from the national mortality statistics provided by the Hellenic Statistical Authority (ELSTAT). Data included all deaths due to infectious diseases that occurred in Greece from 2001 to 2016, by sex, age, region of residence, and cause. Data on Gross Domestic Product (GDP) and unemployment was also derived from the national statistics of ELSTAT, while data on regional public hospital expenditure were provided by the Ministry of Health. For international comparisons, data from the European WHO Health Information Gateway were used [17].

Cause of death classification - quality control of the data

Causes of death were classified in eight groups according to International Classification of Diseases (ICD): (i) intestinal infectious diseases (ICD-9 codes 001-006, ICD-10 codes A00-A09); (ii) tuberculosis (ICD-9 codes 010-018, ICD-10 codes A15-A19); (iii) septicemia and other bacterial diseases (ICD-9 codes 020-035, 037-041, ICD-10 codes A30-A38, A40-A49); (iv) viral diseases (ICD-9 codes 045-079, ICD-10 codes A80-A89, B00-B19, B25-B34); (v) other infectious diseases (ICD-9 codes 080-139, ICD-10 codes A50-A79, A90-A99, B35-B99); (vi) meningitis (ICD-9 codes 036, 320-322, ICD-10 codes A39, G00-G03); (vii) pneumonia (ICD-9 codes 480-486, ICD-10 codes J12-J18), and (viii) influenza (ICD-9 code 487, ICD-10 codes J10-J11).

ICD-10 has been implemented in Greece since 2014. As it has been pointed out in the literature, in time series analysis of mortality, the introduction of ICD-10 may lead to abrupt changes in cause-specific mortality rates [18]. To avoid that type of classification issue, data of 2014-2016 were provided by ELSTAT under both classifications, and they were cross-checked by a group of diseases. Effects were detected in the group (iii) “septicemia and other bacterial diseases”, which according to ICD-10 includes “unspecified septicemia, septic shock” (code A41.9), previously classified as “Symptoms, Signs and Ill-defined Conditions” (under ICD-9 code 780), and because of that, they were excluded from the group of “other bacterial diseases” (codes 020-041). Consequently, the introduction of code A41.9 in ICD-10 artificially increased the number of deaths of the group “septicemia and other bacterial diseases” and reduced unspecified conditions. To ensure temporal comparability of data, we used ICD-9 classification for the entire 2001-2016 period, avoiding artificial jumps in mortality during the last two years. No effect was detected in the other groups of diseases.

Hellenic Statistical Authority applies on the data the validation and quality management processes which have been defined by the statistical office of the European Union (Eurostat). In addition, the quality control of the data used in the present study included crosscheck of sex-and-age subtotals by an ICD code. Excluding the aforementioned classification issue, no other problem was detected in the dataset.

Indicators

For each group of causes, age-specific mortality rates and sex-and-age Standardized Death Rates (SDRs) were calculated, with direct standardization, using as standard the population of Greece in 2011.

Period identification

Although at the economic level the crisis onset is dated at 2008, major health budget cuts and other austerity measures in healthcare were applied in Greece in 2011. Crisis effects on GDP and unemployment were gradually manifested since 2009, but the greater impact was shown since 2011. Therefore, 2011 was designated as the cut-point between the “pre-austerity” period, and the exposed to austerity 2011-2016 period, the last combining both largely affected socio-economic conditions and large cuts in the health budget.

Data analysis

Annual cause-specific SDRs for the years 2001-2016 were calculated. The impact of the economic crisis on SDRs was tested with Interrupted Time Series analyses (ITS) using an autoregressive integrated moving average model (ARIMA 1,0,0). Then, adopting a quasi-experimental approach, the cumulative SDRs of the “exposed to austerity” period (2011-2016) and of an equal “non-exposed to austerity” period before 2011 (2005-2010) were calculated. The cumulative SDRs were compared by calculating Standardized Rate Ratios (SRR) and their 95% confidence intervals (CI). In addition, age-specific mortality rates for each period were calculated. The regional variation of mortality was explored by calculating annual and cumulative SDRs by region.

Social determinants

Using Pearson’s and Spearman’s correlation coefficients, the association of mortality rates with GDP per capita, unemployment rate, long-term unemployment rate (over 12 months) and public hospital expenditure per capita was explored. This analysis was based on the regional annual values of all variables, achieving a total of 208 observations of each variable. Statistical analyses were performed with SPSS V24 (IBM Corp., Armonk, NY, USA).

## Results

**Figure 1:**
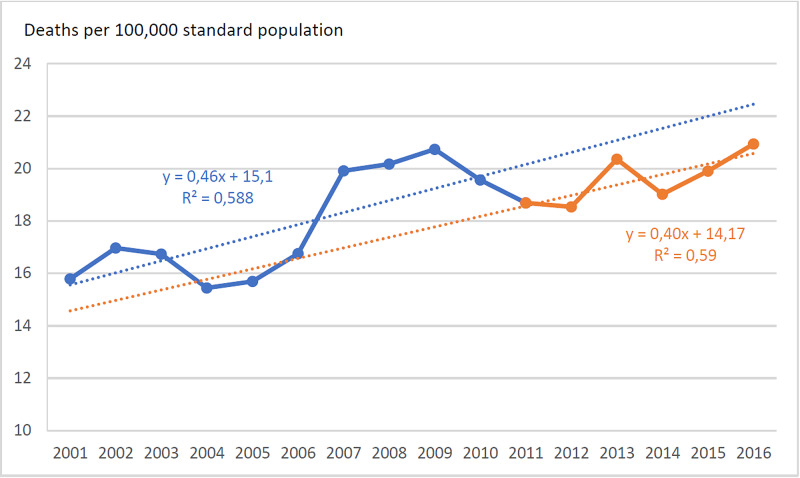
Annual Standardized Death Rate of Infectious Diseases, Greece, 2001-2016

**Figure 2:**
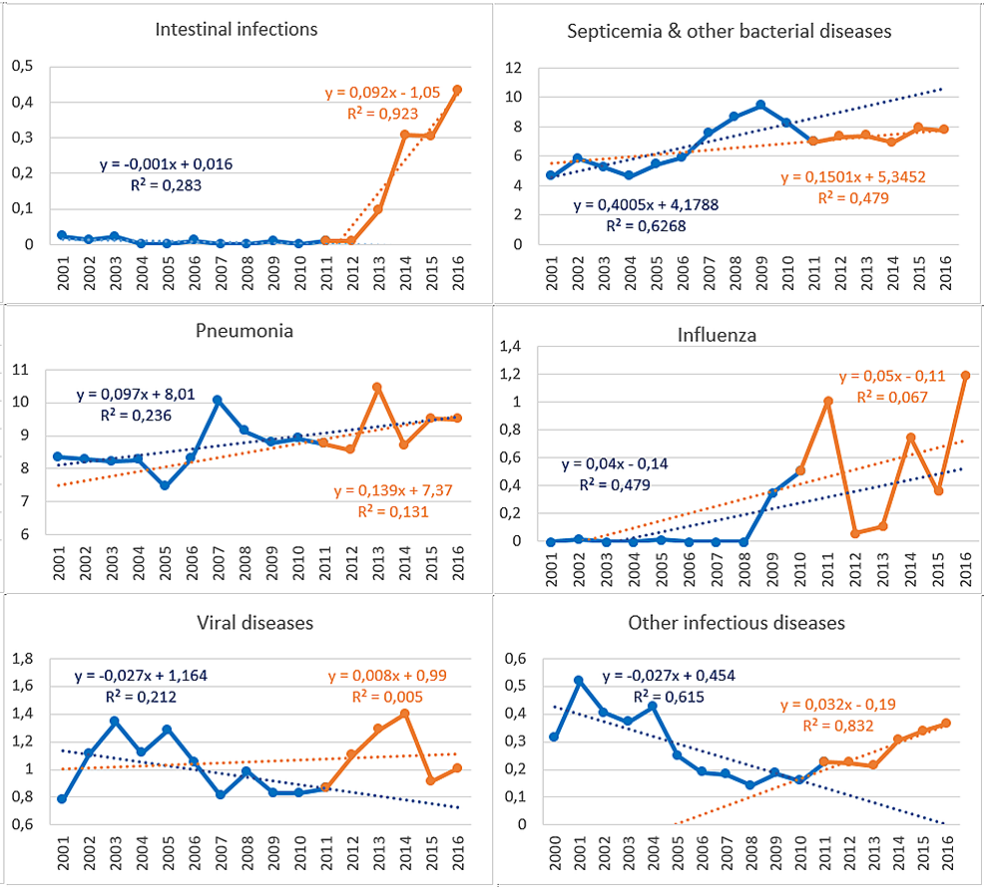
Cause-Specific SDR of Infectious Diseases, 2001-2016 (Deaths per 100,000 Standard Population) SDR: standardized death rate

During 2001-2016, 30,575 deaths due to infectious diseases occurred in Greece, 44.0% of them in 2011-2016. Deaths due to pneumonia count for 47.5% of all deaths. Septicemia and other bacterial diseases count for 37.2%, septicemia being the dominant cause of this cluster at a percentage of 98.5%. Figure 1 displays the trend of the overall annual SDR, while Figure 2 displays the trends of the cause-specific SDRs. Testing with ITS analysis the impact of the crisis on the annual SDRs, a significant effect was found in intestinal infections (t=11.6, p<0.001) and other infectious diseases (t=3.8, p<0.01) but not in the other disease groups.

Comparing the exposed to austerity period 2011-2016 with the non-exposed 2005-2010, the overall SDR increased by 5.0% (95% CI 2.4%-7.7%) (Table 1). The cause-specific SDR recorded a significant increase in intestinal infections, viral diseases, pneumonia, influenza and other infectious diseases. Conversely, a significant decline was observed in the SDR of tuberculosis (TB) and meningitis and no effect on septicemia.

**Table 1:** Standardized Death Rate (SDR) of Infectious Diseases and Standardized Rate Ratio (SRR), Before and After Economic Crisis, by Group of Infectious Diseases

	No of deaths 2005-2010	No of deaths 2011-2016	±(%)	6-year SDR 2005-2010	6-year SDR 2011-2016	SRR	95% CI of SRR	
Intestinal infectious diseases	2	137	6750%	0.0	1.3	58.0	14.4	234.1
Tuberculosis	501	368	-26.5%	5.1	3.3	0.7	0.6	0.8
Septicemia & other bacterial diseases	4,539	5,088	12.1%	47.3	46.7	1.0	0.9	1.0
Viral diseases	601	742	23.5%	5.9	6.7	1.1	1.0	1.3
Other infectious diseases	116	190	63.8%	1.1	1.7	1.5	1.2	1.9
Meningitis	244	179	-26.6%	2.3	1.6	0.7	0.6	0.8
Pneumonia	5,277	6,366	20.6%	54.8	58.3	1.1	1.0	1.1
Influenza	94	389	313.8%	0.9	3.5	4.0	3.2	5.0
Total	11,374	13,459	18.3%	117.4	123.2	1.1	1.0	1.1

**Figure 3:**
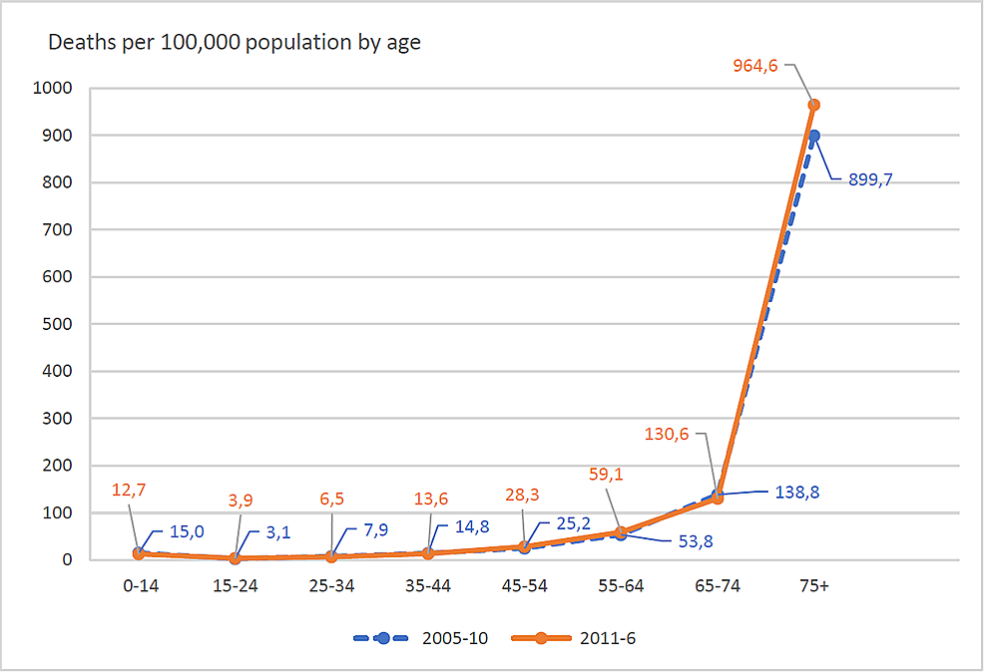
Age-Specific Mortality Rates 2005-2010, 2011-2016.

Age-specific mortality rates (Figure 3) exhibited a significant increase in age over 75 years (p<0.001), while the changes in all other age groups were found not significant. The disease groups recording significant mortality increase in the age group over 75 years old were intestinal infection, pneumonia, and influenza (p<0.001)

Exploring the trends in SDR of infectious diseases in the European countries most affected by the crisis (Table 2), notable differences were observed. In Italy, Greece, Hungary, and Lithuania, the post-crisis SDR showed an upwards trend; in Ireland and Spain, a downwards trend; while in Portugal, Poland, and Estonia, no significant change was recorded.

**Table 2:** Standardized Death Rate of Infectious Diseases in Selected European Countries Affected by the Economic Crisis, 2005-2015 (*) Data include deaths due to infectious and parasitic diseases (A00-A99, B00-B99), meningitis (G00-G03), influenza (J10-J11), and pneumonia (J12-J18). (-) Adequate data not available. Source: WHO Regional Office for Europe. WHO European Health Information Gateway [17].

	Ireland	Portugal	Spain	Italy	Greece	Hungary	Poland	Estonia	Lithuania
2005	44.8	(-)	25.2	15.3	9.5	11.8	26.4	19.1	32.3
2006	44.1	(-)	21.6	13.6	9.8	9.3	25.6	21.8	32.5
2007	28.1	42.5	22.6	14.1	11.6	9.9	24.9	19.4	35.1
2008	31.2	44.5	22.4	13.8	11.4	8.7	25.7	17.6	34.6
2009	31.2	43.8	20.0	14.1	11.7	9.0	26.3	20.2	28.5
2010	26.3	41.5	17.4	13.9	10.8	9.3	25.2	16.2	22.9
2011	23.3	39.3	17.8	15.9	10.4	10.4	26.2	17.7	24.8
2012	23.2	44.0	18.1	17.1	10.0	11.9	26.1	18.1	25.6
2013	21.1	40.1	16.2	16.5	12.1	11.9	28.8	20.0	31.1
2014	(-)	36.7	16.8	16.2	19.3	11.2	25.5	21.3	27.8
2015	(-)	(-)	18.7	19.4	20.0	11.7	29.0	18.8	30.8

Exploring the correlation of the overall mortality rate with the aforementioned socio-economic variables (Table 3), a positive association was found with GDP per capita, unemployment, and long-term unemployment, and conversely, a negative association with hospital expenditure. Similar correlations were found in mortality of septicemia and pneumonia. A negative association with GDP was found in mortality of intestinal infections and viral diseases. Unemployment and long-term unemployment were positively correlated with mortality of intestinal infections and viral diseases, but negatively with mortality of tuberculosis and meningitis.

**Table 3:** Correlation Analysis Results (Correlation Coefficients and P Values) GDP: gross domestic product

		Intestinal infections	Tuberculosis	Septicemia	Viral diseases	Pneumonia	Influenza	Meningitis	Other infect. diseases	Total
GDP per capita	Pearson's r	-0.19	-0.06	0.35	-0.14	0.11	-0.09	0.08	-0.10	0.30
	p	0.005	0.38	<0.001	0.04	0.11	0.37	0.26	0.14	<0.001
	Spearman's ρ	-0.28	-0.11	0.39	-0.21	0.10	-0.01	0.12	-0.13	0.31
	p	<0.001	0.13	<0.001	0.002	0.156	0.94	0.08	0.07	<0.001
Unemployment	Pearson's r	0.55	-0.29	0.26	0.08	0.29	0.02	-0.20	-0.06	0.34
	p	<0.001	<0.001	<0.001	0.25	<0.001	0.82	0.003	0.38	<0.001
	Spearman's ρ	0.53	-0.30	0.26	0.05	0.20	-0.02	-0.22	-0.10	0.29
	p	<0.001	<0.001	<0.001	0.46	0.004	0.88	0.001	0.16	<0.001
Long-term unemployment	Pearson's r	0.60	-0.29	0.23	0.17	0.32	0.07	-0.19	-0.04	0.34
	p	<0.001	<0.001	0.001	0.02	<0.001	0.49	0.006	0.61	<0.001
	Spearman's ρ	0.56	-0.28	0.21	0.17	0.24	0.06	-0.19	-0.08	0.27
	p	<0.001	<0.001	0.003	0.02	<0.001	0.55	0.008	0.26	<0.001
Hospital expenditure per capita	Pearson's r	0.01	-0.02	-0.39	-0.07	-0.35	0.11	-0.12	0.27	-0.46
	p	0.95	0.89	0.004	0.65	0.011	0.43	0.39	0.06	0.001
	Spearman's ρ	-0.03	0.06	-0.31	-0.06	-0.30	0.15	-0.07	0.23	-0.42
	p	0.86	0.68	0.025	0.68	0.03	0.28	0.60	0.11	0.002

## Discussion

In this study, we explored the trends in mortality of infectious diseases in Greece to investigate whether financial crisis affected mortality rates in the general population. The comparison of the overall SDR between the exposed and non-exposed to austerity period showed a statistically significant increase by 5%, indicating an adverse impact of recession and austerity on infectious disease mortality. Similar findings with pro-cyclical effects have been reported in certain European countries [13,14], while no effect [16], or counter-cyclical effects were found in others [15]. Exploring the literature about the impact of economic downturns on mortality of infectious diseases, the evidence is mixed. An ECDC report of 2013 [2] and a systematic review of 2011 [7] reported a number of studies finding significant associations of the incidence of communicable diseases with poverty and unemployment, indicating that the social effects of financial crises are associated with a rise in communicable diseases, and conversely, a number of other studies finding no association or negative association with the same variables. Several studies provide evidence that economic downturns may affect specific population groups, such as children [9,10], unemployed [7], or migrants [11], but the evidence about the effects on the general population is limited. Moving from morbidity to mortality, the evidence is more weak and contradictive. Older studies suggested that mortality of infectious diseases probably decreases during recessions [19]. On the contrary, some authors recently suggested that the last recession of 2008 may have caused a rise in certain risky behaviors, increasing the risk of transmission [2,5,9]. However, findings from other studies do not support such a hypothesis [15,16]. It seems more reliable that the impact differs between populations, depending on their epidemiologic characteristics, risk factors, and the specific effects of the financial crisis on each one [4].

Exploring the association of the overall mortality rate with the various socio-economic variables, unemployment and hospital expenditure were found to be the most consistent determinants, displaying significant association with the overall and many of the cause-specific mortality rates. The impact of unemployment on infectious diseases has been confirmed in several studies [7,20]. Although the causal mechanisms are not clear enough, it has been proposed that they are related to greater exposure of unemployed to conditions increasing the risk of transmission [7,20] and to the reduced affordability for appropriate treatment [4,20]. In addition, in Greece and other countries with a Bismarck-type health insurance system, health benefits are directly associated with employment status; long-term unemployment results in a loss of health benefits and consequently to a restriction of access to healthcare by the unemployed and their families [21]. When unemployment is rising and becomes long-standing, this feature plays a critical role. The linkage of health insurance benefits with unemployment may explain a part of the differences observed between countries with different health systems. Regarding hospital funding, the negative correlation of mortality with hospital expenditure suggests that a part of that mortality is avoidable through appropriate and timely healthcare [22]. Adequate funding increases healthcare effectiveness and contributes to achieving better health outcomes. In addition, this correlation indicates that regional inequalities in mortality are associated with funding inequalities. Hospital funding may have different significance for the control of the various infectious diseases and therefore it can be differently correlated with the cause-specific mortality trends.

On the other hand, a positive association of the overall mortality rate with GDP was recorded, implying that recession may have had a favorable impact on mortality rates. However, GDP was found to be negatively associated with certain cause-specific mortality rates (intestinal and viral infections), and conversely, positively associated with others (septicemia and other bacterial diseases). It has been noticed that the significance of GDP as a predictor of the risk of infectious disease is found disease-dependent [20]. Therefore, the direction of the GDP correlation with the overall infectious disease mortality in each country or population depends on the case-mix of infectious diseases.

Pneumonia is the cause of the largest cluster of mortality in our study, however, with a rate among the lower of Europe [17]. Mortality of pneumonia was found higher during the austerity period and positively correlated with the rising unemployment and health budget cuts. The positive association with unemployment, although does not accord with the results of certain studies [19], is probably due to the aforementioned direct linkage of unemployment with access to healthcare, as well as a possible worsening of living standards and hygiene conditions due to unemployment. The negative correlation with hospital expenditure supports that inadequate healthcare funding may result in a worsening of pneumonia outcomes.

Mortality from influenza, which was found fourfold during 2011-2016, began increasing in Greece since 2009 when the pandemic strain H1N1 was spread in the country. This fact does not allow attributing the mortality rise to austerity, because it is determined by epidemiological factors and possibly by a fluctuation in weather conditions. The hypothesis that austerity measures may have affected the sound implementation of the anti-influenza vaccination programs is not supported by the facts, since there is no evidence that anti-influenza vaccination has been neglected during the past years [15,23].

Septicemia represents the second biggest cluster of infectious disease mortality in Greece. The case fatality rate of sepsis has decreased in most European countries [23], mainly due to improvements in treatment and prognosis [24]. The study findings show that mortality of septicemia was not adversely affected by the financial crisis. However, mortality rates were negatively correlated with hospital expenditure. Considering that treatment of septicemia is mostly a matter of hospital care, hospital underfunding may affect its effectiveness [22].

Tuberculosis is typically considered as a disease strongly associated with poverty and living standards, displaying higher rates in socially and economically disadvantaged populations [25], which are often those with the poorest access to healthcare [2]. During the past decades, a great reduction in mortality of TB was achieved in most European countries [26]. Following these trends, Greece recorded a remarkable decline, reaching at a rate almost half of the average EU rate [17,26], which was continued after the crisis onset. This is probably due to the long-term effectiveness of the overall anti-TB strategy of the country, which reduced the risk for a disease rebound [25].

Mortality due to viral diseases is negatively associated with GDP and positively with unemployment. This group includes deaths due to hepatitis at a percentage of 78.3%, and to all other viral diseases at 21.7%. The cumulative SDR of hepatitis remained unchanged between the two periods (5.1 vs. 5.0/100,000), while the SDR of all other conditions doubled from 0.9 to 1.9/100,000. A part of the increase is probably due to the emerging West Nile virus outbreak, which first occurred in Greece in 2010 and thereafter established in the country [27].

Mortality from intestinal infections recorded a sharp and significant increase mainly in the elderly, with remarkable regional variation. Further exploring the causes of death in this cluster, it was found that 86.2% of them were due to protozoal and viral intestinal diseases, which are less related to hospital-acquired infections. Intestinal infections are related to living standards, poor hygiene, and general public health conditions [2]. According to WHO, the mortality due to intestinal infections in most of the Southern European countries during the austerity period was significantly correlated with GDP and unemployment [28]. Our findings also showed a strong positive correlation with unemployment and long-term unemployment, and a negative association with GDP. Although the seasonal character of these diseases may cause a large fluctuation in their mortality rates [14], the significant association with the worsening of socio-economic conditions supports that probably it has been affected by the crisis. The causal mechanisms may be associated with worsening of living standards and hygiene conditions due to unemployment and poverty.

Mortality due to meningitis includes cases of meningococcal and non-meningococcal bacterial infections, other infectious meningitides, and cases of unspecified cause. Meningococcal infections count for only 0.6% of this group, obviously because of the introduction of the new vaccines for A, C, Y, W-135 serogroups in the national vaccination programs, as well as the availability of the vaccine against serogroup B for special population groups [29]. The group of other or unspecified meningitides includes cases mainly due to *Streptococcus pneumoniae*, *Hemophilus influenza*, *Listeria monocytogenes*, and *Streptococcus agalactiae* [30]. The decline in SDR due to these types of meningitis is probably related to the implementation of vaccination programs against *Hemophilus influenzae* B and *Streptococcus pneumoniae*, which had a significant contribution in mortality reduction of these diseases [30]. As is already mentioned, the sound implementation of the national vaccination programs has not been considerably affected by health budget cuts or other austerity measures [15,23].

Limitations

The nature of the data and the design of this study set several limitations. Aggregated mortality data do not allow exploring the association of individual deaths with healthcare performance and public health conditions, or calculating incidence and case-fatality rates. The study design does not permit identifying the causal mechanisms of the effects. The mortality of infectious diseases is determined by several factors, which raise difficulties in the interpretation of changes in mortality rates. All these factors coincide with the impact of socio-economic variables, raising difficulties when attempting to isolate the effects of the socio-economic developments on mortality rates.

## Conclusions

The study findings indicate that the overall mortality of infectious diseases in Greece was adversely affected by the economic crisis. The effects were found to be disease-dependent. Mortality increase was observed in intestinal infections, pneumonia, influenza, viral diseases and other infectious diseases, while a reduction was observed in tuberculosis and meningitis and no effect in mortality of septicemia. Unemployment and hospital expenditure were found to be the main socio-economic determinants, while GDP exhibited controversial associations. Intestinal infections, pneumonia, and viral diseases were found to be more affected by unemployment, while pneumonia, septicemia, and intestinal infections more affected by cuts in hospital funding. The causal mechanisms of the impact remain unclear, requiring further research. Broader implementation of case notification and a linkage with mortality statistics may provide more opportunities to take advantage of routine data to investigate factors affecting mortality from infectious diseases.

The study findings have public health implications in identifying groups at greater risk during a crisis and in planning appropriate interventions to protect people’s health. Resource allocation and hospital funding should take into account the risk of adverse effects in certain disease categories such as pneumonia and septicemia. Unemployed and their families face greater risk and must be protected with specific programs and interventions. This need is more critical when unemployment is associated with the loss of health insurance benefits and because of that, with reduced access to healthcare. Furthermore, exacerbation of socio-economic conditions may increase the risk of emerging outbreaks or the risk of inadequate response to them, requiring public health readiness and infectious disease surveillance.
